# People with intellectual disability and their risk of exposure to violence: Identification and prevention – a literature review

**DOI:** 10.1177/17446295241252472

**Published:** 2024-05-07

**Authors:** Mikaela Starke, Anneli Larsson, Elisabeth Punzi

**Affiliations:** 3570 University of Gothenburg, Sweden

**Keywords:** persons with intellectual disabilities, risk of exposure to abuse and violence, identification and prevention of abuse and violence, literature review

## Abstract

The aim of the literature review was to identify knowledge and knowledge gaps concerning risks of violence toward children, youth, adults and elderly with intellectual disabilities, and how risks can be identified and prevented. The research revealed that children, youths and adults labelled with intellectual disabilities are more exposed to violence than others and that the target group lack knowledge about risks of violence and what it means to be exposed to violence. It was also found that professionals who work with people with intellectual disabilities may lack knowledge about violence, and those who work with violence lack knowledge about intellectual disabilities. There is thus a need to further elaborate routines to identify exposure to violence, and to identify the target group and a need to create collaborative teams with professionals who have in-depth knowledge of violence, and those who have in-depth knowledge about the target group.

## Background

The United Nations Convention on the Rights of Persons with Disabilities (United Nation, 2006) states in Article 16 that “States Parties shall take all appropriate legislative, administrative, social, educational and other measures to protect persons with disabilities, both within and outside the home, from all forms of exploitation, violence and abuse, including their gender-based aspects” (UNCDPR). Violence against persons with disabilities is a violation of human rights and thus an essential societal issue. The World Health Organization (WHO) defines violence in the “World report on violence and health” (WRVH) as ”The intentional use of physical force or power, threatened or actual, against oneself, another person, or against a group or community, that either results in or has a high likelihood of resulting in injury, death, psychological harm, maldevelopment, or deprivation.” (World Health Organization).

The terms abuse and violence are related concepts but have distinct meanings and implications. It is crucial to address both abuse and violence, as they can have severe consequences on individuals and communities. Abuse refers to maltreatment or misuse of power, control, or a position of trust to harm or exploit another person emotionally, psychologically, physically, or sexually (Follingstad 2007). It takes various forms, such as verbal, emotional, financial, social abuse or neglect by withholding essential needs of a person purposeful or unintentional (Robinson and Chenoweth 2012). Abuse is commonly defined as a pattern of behaviour used by one person to gain and maintain power and control over another (Dutton 2012). A comprehensive definition of violence includes four elements: behavior that is (a) intentional, (b) unwanted, (c) nonessential, and (d) harmful (Hamby 2017). It often involves a direct confrontation or act of aggression and typically refers to the intentional use of physical force or power with the aim of causing harm, injury, or death (Mider 2013).

The present review comprises both terms since violence often is a component of abuse, but not all abuse involves physical harm or violence. Abuse is more insidious and may involve a pattern of behaviour that undermines a person over time, whereas violence is often an immediate, observable act. Violence is a more specific term that typically involves physical force, whereas abuse is a broader term, encompassing various forms of harm and mistreatment.

Abuse and violence toward persons with intellectual disabilities has for several decades been highlighted as a global problem and several investigations have resulted in recommendations and reforms. Despite efforts to prevent and reduce violence, there is still an increased risk of exposure to violence among persons with disabilities (Adams and Erevelles, 2017; Banks et al. 2017; Mikton et al. 2014; Tomsa et al. 2021), and persons with disabilities, across the world, have a greater risk of being exposed to violence and abuse compared to citizens without disabilities (Baladerian et al. 2013).

Persons with intellectual disabilities are specifically at risk (Stone 2018). They also have difficulties reporting abuse as they cannot make their voices heard in the legal systems as others can and are more dependent on formal and/or informal caregivers (Didi et al. 2016). Risks may be lowered if persons with intellectual disabilities receive interventions aimed at decision-making in situations where there is a risk of abuse. In a randomized controlled trial, Hickson et al. (2015) found that adults who received such an intervention improved their capacity for effective decision-making in abuse situations. Moreover, young people with intellectual disabilities who are transitioning into adulthood should receive sex education, since such education counteracts the risk for sexual transgressions (Wilson and Frawley 2016).

The continuous reports about exposure to violence and abuse motivates the need to compile research about the risk of violence and abuse, and the identification and prevention of it, to identify knowledge gaps. During recent years, several scoping and systematic reviews have investigated violence and abuse toward persons with intellectual disabilities (Meyer et al. 2022). Most previous reviews specifically concern adults with intellectual disabilities (Collins & Murphy 2022; Gilloway et al. 2020; McNally et al. 2021; Stobbe et al. 2021). There is a need to investigate children and youths and the specific risks they are facing such as witnessing partner violence between adults (Bowen and Swift 2019; Ravi and Black 2022). Therefore, a life course perspective is beneficial. This can move the field towards actual change for those affected. Accordingly, the aim of this literature review was to identify knowledge and knowledge gaps concerning risks of violence and abuse toward children, youth, adults and elderly with intellectual disabilities, and how risks can be identified and prevented. This review can provide findings that answers the following research questions:

What is known about risks of exposure to violence and abuse for persons with intellectual disabilities?

How can risks of violence and abuse for persons with intellectual disabilities be identified and prevented?

## Method

A literature review was chosen as it offers a flexible way of working that suits this study’s broad research questions. We were however inspired by the procedure of scoping review and the methods employed followed the guidance by Armstrong et al. (2011): preparation, searches, assessment of relevance, mapping, and compilation. The review also used the PRISMA-ScR framework (Tricco et al. 2018) to structure the results. This ensures methodological transparency of the findings and increases the relevance for decision making.

### Preparation; inclusion and exclusion criteria

To prepare and decide on inclusion and exclusion criteria the structured format of SPIDER, an abbreviation of Sample, Phenomenon of Interest, Design, Evaluation and Research type, was used (Cooke et al. 2012).

To be included, studies had to:

1. include all ages of persons with intellectual disabilities.
2. be based on data derived from the target group, parents, professionals or others with a focus on persons with intellectual disabilities.
3. focus on abuse/violence.
4. specify the type of abuse/violence that was in focus.
5. be an original study of any design and research type.
6. be published between 2000 and May the 2^nd^ 2022.
7. be written in English, Norwegian, Danish, or Swedish.
8. be published in a peer review journal.

The studies that were excluded:

1. analyzed intellectual and other disabilities without specifying differences between them.
2. did not specify the type of abuse/violence.
3. were published before 2000.
4. were written in another language than English, Norwegian, Danish, or Swedish.
5. were not original, empirical studies.
6. were published in journals were not recognized as peer review journals.

### Literature search strategy

The search strategy was developed in consultation with librarians at the University of Gothenburg, Sweden using the following databases: Scopus, Sociological Abstracts, Applied Social Sciences Index and Abstracts (ASSIA), Social Service Abstracts, the International Bibliography of the Social Sciences (IBSS), the Criminal Justice Database and PsycINFO.

Even though the term intellectual disabilities is most often used, it should be noted that researchers use other terms for the same or similar population such as learning and developmental disabilities, impairment, learning difficulty are used. Therefore, other terms than intellectual disabilities were included as search terms. For *Sample*, the search terms were: “intellect* disabilit*” OR “intellectual developmental disabilit*” OR “intellect* dysfunct*” OR “intellect* impairment*” OR “cognit* disabilit*” OR “cognit* difficult*” OR “learning difficulty*”. In the PsycINFO database, neither the authors or the librarians could use the search term “learning difficult*”. The search terms that were chosen for *Phenomenon of Interest* were: violenc* OR harm OR abus* OR neglect* OR maltreat* OR honor* OR mutilation* OR force* OR prostitution* OR traffic* AND “Expos* to risk” OR “risk* situation*” OR risk* OR threat* OR danger* OR “Recognis* risk*” OR “indent* risk*” OR “prevent* risk*”. The search term abus* covers various forms of abuse, including sexual abuse. Specific search terms for prostitution and trafficking were added since they are not necessarily covered by the term abuse.

No search terms concerning design, evaluation or research type were used and all types of different study designs, evaluations and research types were included. All database searches included all search words listed above and were searched for in the full text field. The findings were downloaded into End Note 20.

### Assessment of relevance

The process of assessment included the following steps:

1. First author deleted all duplicates in End Note.
2. First author screened title and abstract against the exclusion and inclusion criteria.
3. The identified articles were read in full text, all read by the first author and half of them by the second author and half of them by the third author.
4. All authors read the remaining articles in full-text and assessed them using a protocol based on SPIDER and the inclusion criteria.
5. A joint decision on included articles was made.

### Mapping

**Figure 1.**
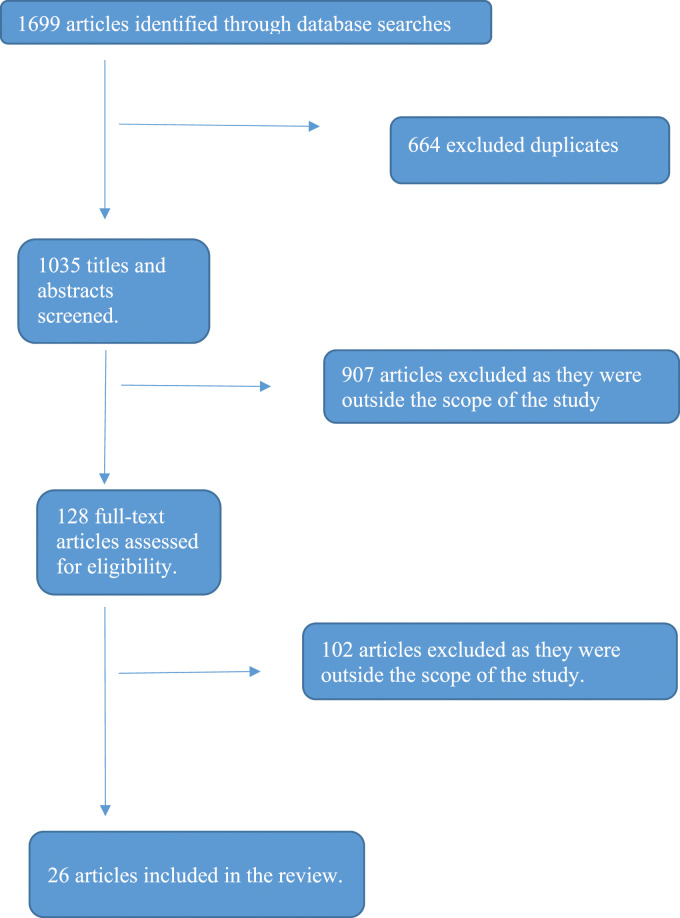
The results of the search in the different databases.

The screening process is represented in a PRISMA diagram (Tricco et al. 2018) in Figure 1. The 128 full-text articles that were assessed for eligibility were all read by the first author and 64 of them by the second author and 64 of them by the third author. If there was disagreement, all three researchers read and commented. There needed to be a shared agreement for the article to be included. The 26 articles that were included were read by all three authors to have a decision in consensus.

### Compilation

In this last step, conclusions are drawn from the results, and knowledge gaps are presented. First a descriptive analysis of each article (n=26) was compiled, based on the findings from each full text presented in Table 1 and summarized by author/s, journal, year of publication, country in which the research was done, sample, phenomena of interest and type of study.

**Table 1.** The articles included in the literature review.

Author/s and title	Journal	Year	Country	Sample	Phenomena of interest	Type of study
**Almack et al.** Parental Negotiations of the Moral Terrain of Risk in Relation to Young People with Intellectual Disabilities	Journal of Community & Applied Social Psychology	2009	UK	Parents’ experiences of transition from child with intellectual disabilities to adult services.	To examine how parents negotiate boundaries and position in relation to risk concerning their child with intellectual disability and their transition into adulthood.	Interview study adopting a narrative approach.
**Carrellas et al**. Sexual victimization and intellectual disabilities among child welfare involved youth	Child Abuse & Neglect	2021	USA	334 youth (aged 18–19.5) from a nationally representative sample of 5,872 youth involved in child welfare.	To explore the risk and protective factors of youths with intellectual disabilities associated with experiencing sexual victimization and engagement in transactional sex who are transitioning out of child welfare services.	Secondary analysis of a subset of data from the National Survey on Child and Adolescent Well-Being.
**Clawson & Fyson** Forced marriage of people with learning disabilities: a human rights issue	Disability & Society	2017	UK	An interview with nine interviewees, representing professionals and a non-governmental organization consultant. An online survey completed by 287 professionals.	An exploratory study of forced marriage of people with learning disabilities, identify both similarities and differences between forced marriage of people with learning disabilities and other forced marriages.	Interview and survey study
**Clawson et al.** The demographics of forced marriage of people with learning disabilities: findings from a national database	The Journal of Adult Protection	2020	UK	The data includes a total of 593 cases where a disability was identified, of which 554 cases (93%) related to someone with learning disabilities.	The aim was to compare the UK demographics of forced marriage of people with learning disabilities and people without learning disabilities.	Register study
**de Mello et al.** Dialogic Feminist Gathering and the Prevention of Gender Violence in Girls With Intellectual Disabilities	Frontiers in Psychology	2021	Spain, Brazil	Dialogic Feminist Gatherings (DFG) were implemented in a special school located in Valencia during the 2018–2019 and 2019–2020, including a group of 19 non-mixed female students, seven female teachers, and the mother of one of the students.	To analyze the Dialogic Feminist Gatherings (DFG) and its transfer and impact on adolescent girls with intellectual disabilities.	Two in-depth interviews, analysis of the field diary of 24 intervention sessions and a focus group with teachers.
**Fanslow et al.** Lifetime Prevalence of Intimate Partner Violence and Disability.	American Journal of Preventive Medicine	2021	New Zealand	Out of 2888 persons aged ≥16 years, 524 reported having ≥1 disability, of whom 309 (58.9%) reported having multiple disabilities.	To compare lifetime prevalence of intimate partner violence (physical, sexual, psychological, controlling behaviors, and economic abuse) for people with different types of disabilities (including intellectual disability) and those without disabilities.	A Population-Based cross-sectional study using face-to-face interviews
**Helton et al.** Sexual Abuse of Children With Learning Disabilities	Child Maltreatment	2018	USA	2033 children over the age of 4 years.	To examine the sexual abuse profiles of children with learning disability compared to children without learning disability, the inter-connectedness of disability, gender, and maltreatment, differences in level of harm, types of sexual assaults, duration, and perpetrator type.	Register study with data from the second National Survey of Child and Adolescent Well-Being, children who have been in contact with the child welfare system due to an allegation of some type of maltreatment
**Hughes et al.** “I really want people to use our work to be safe” Using participatory research to develop a safety intervention for adults with intellectual disability	Journal of Intellectual Disabilities	2020	USA	A community advisory board, two men and four women, who self-identified as having an intellectual disability and were active in disability rights activities. A national advisory board, three people with intellectual disability who were each paired with a known mentor from the disability rights community. A research team included academics, service providers, a local advisory board, and a national advisory board.	To describe the development and report the results of a feasibility test of the Safety Class.	An intervention and implementation study of the Safety Class
**Kahonde & Johns** Knowledge, perceptions and experiences of risk to sexual violence among adults with intellectual disabilities in Cape Town, South Africa	African Journal of Disability	2022	South Africa	18 women and 9 men with mild to moderate intellectual disability (aged 20–47).	To explore and describe the knowledge and awareness of risk to sexual violence among adults with intellectual disabilities and their perceptions and experiences of risk.	Focus group study
**Kildahl et al.** Clinicians’ retrospective perceptions of failure to detect sexual abuse in a young man with autism and mild intellectual disability	Journal of Intellectual & Developmental Disability	2020	Norway	Five team members with long experience in specialist mental health services for people with autism and/or intellectual disability. They had different professions and roles and relationships with the patient. A face-to-face, in-depth, individual, semi-structured interview.	To explore clinicians’ perceptions of their failure to detect abuse.	Interview study adopting an interpretative phenomenological analysis.
**Ledingham et al.** Sexual Violence Against Women With Disabilities: Experiences With Force and Lifetime Risk	American Journal of Preventive Medicine	2022	USA	A total sample of 16176 female respondents (18−44 years) including women with cognitive disabilities	To analyze the relationship between disability status, including cognitive disabilities and experience of sexual violence.	Cross-sectional study data from the National Survey of Family Growth, a representative in-person survey on a multistage probability sample of non-institutionalized men and women.
**Lin et al.** Domestic violence against people with disabilities: Prevalence and trend analyses	Research in Developmental Disabilities	2010	Taiwan	National data (2006-2009) from “Domestic Violence Report System” and reports on the rate of domestic violence in Taiwan.	To provide a provisional profile of people with disabilities (including persons with intellectual disability), experienced domestic violence.	Register study.
**MacLean et al.** Maltreatment Risk Among Children With Disabilities	Pediatrics	2017	Australia	All children born in Western Australia (1990 -2010), 524,534 children in the population cohort.	To report the prevalence of different disabilities (including children with intellectual disability) within the child protection system and to assess the risk of maltreatment in various types of disability.	Population based register study.
**McCarthy et al.** Risk of forced marriage amongst people with learning disabilities in the UK: Perspectives of South Asian carers	Journal of Applied Research in Intellectual Disabilities	2020	UK	22 South Asian family carers of a family member with learning disabilities 20 women and 2 men.	To report the perspectives of South Asian family carers of adults with learning disabilities.	Interview and focus groups study
**McConnell et al.** Parental cognitive impairment and child maltreatment in Canada	Child Abuse & Neglect	2011	Canada	11562 child maltreatment investigations.	To examine outcomes for children of parents with cognitive impairment in child maltreatment cases.	Secondary analysis of the Canadian Incidence Study of Child Abuse and Neglect core data.
**Majeed-Ariss et al.** The disproportionately high prevalence of learning disabilities amongst adults attending Saint Marys Sexual Assault Referral Centre	Journal of Applied Research in Intellectual Disabilities	2020	UK	Clients aged 18 years or more attending Saint Mary's Sexual Assault Referral Centre in the context of an acute rape or sexual assault.	To identify the prevalence of learning disabilities amongst adult clients attending the Centre and ascertain the similarities/ differences amongst clients with learning disabilities as compared to those without.	A retrospective observational case–control study design.
**Meer & Combrinck** Help, harm or hinder? Non-governmental service providers’ perspectives on families and gender based violence against women with intellectual disabilities in South Africa	Disability & Society	2017	South Africa	Service providers recruited from 17 disability rights organizations, for women with disabilities (*n*=34). Service providers (*n*=24), recruited from 16 organizations dealing with gender-based violence. In total 58 service providers from non- governmental organisations.	To describe the service providers and their reflections about the intricacy of familial relationships for women with intellectual disabilities in South Africa who experienced gender-based violence.	In-depth interview study
**Nixon et al.** Estimating the risk of crime and victimisation in people with intellectual disability	Social Psychiatry and Psychiatric Epidemiology	2017	Australia	A randomly extracted community comparison sample of 4830 individuals aged 18–65, from the Victoria, Australian Electoral Commission database. Criminal charges and reports of victimization were compared to a non-disabled community comparison sample (*n* = 2085).	To estimate the prevalence of criminal histories and victimization, using a large, well-defined sample of people with intellectual disability, and compare this with a community sample without intellectual disability.	A case-linkage study including people with intellectual disability registered in disability services whose personal details were linked with a state-wide police database.
**Northway et al.** How do people with intellectual disabilities view abuse and abusers?	Journal of Intellectual Disabilities	2013a	UK	Individual interviews (n=14) and seven focus groups, involving 47 people; 19 ere women and 27 men with intellectual disability.	The aim was to explore people with intellectual disability and their views about abuse.	An interview and focus group study.
**Northway et al.** Keeping Safe and Providing Support: A Participatory Survey About Abuse and People With Intellectual Disabilities	Journal of Policy and Practice in Intellectual Disabilities	2013b	UK	A questionnaire was distributed to participants with intellectual disabilities attending a residential research event and as a postal survey across Wales. 107 answers.	To examining how people with intellectual disabilities understand abuse, how they can keep themselves safe, and how they can best be supported should abuse occur.	A survey study
**Nyberg et al**. Signs of abuse in children with disabilities: A rapid review with expert panel social validation	Journal of Intellectual & Developmental Disability	2021	Sweden, South Africa	Researchers and professionals (n=39), members of international organizations targeted preventing violence and abuse towards children with/without disabilities, representing different countries.	To identify and socially validate signs of abuse in children with disabilities.	A mixed-method sequential design, including 1. a rapid review of publications about signs of abuse in children with disabilities 2. social validation using an online survey.
**Paquette et al.** Factors associated with intellectual disabilities in maltreated children according to caseworkers in child protective services	Children & Youth Services Review	2018	Canada	The sample consisted of 1021 maltreated children, aged 6–17.	To identify the individual, environmental and service-related factors that distinguish maltreated children with intellectual disabilities from those without intellectual disabilities.	A secondary use of register data derived from the Québec Incidence Study.
**Prendergast et al.** Surprisingly complex life: Sue Leung’s story	Asia-Pacific Psychiatry	2013	Hong Kong, Macao	Sue Leung and her mother and caregiver, Mrs. Leung	To explore the challenges and difficulties a mildly intellectually disabled woman has to face in her life.	Narrative case study
**Robinson & Graham** Feeling safe, avoiding harm: Safety priorities of children and young people with disability and high support needs	Journal of Intellectual Disabilities	2021	Australia	22 children and young people (aged 7–25; males=11, females=11) with intellectual disability and some with multiple disabilities.	To explore what helps and constrains children and young people with intellectual disability in being safe in institutional settings.	Mixed methods, individual and small group interviews and customized individual approaches.
**Stevens** Examining Emerging Strategies to Prevent Sexual Violence: Tailoring to the Needs of Women With Intellectual and Developmental Disabilities	Journal of Mental Health Research in Intellectual Disabilities	2012	USA	A follow-up study on a workshop including six key informants in the field of intellectual and developmental disability and sexual violence prevention.	To synthesize information from a workshop and follow-up interviews, examining emerging strategies to prevent sexual violence tailored to the needs of women with intellectual and developmental disabilities.	Phone interview study
**Vered** Associations between Parental Mental Health and Child Maltreatment: The Importance of Family Characteristics	Social Sciences	2021	Israel	The study sample comprised 522 parents (261 mothers and 261 fathers) whose parental rights were terminated due to child maltreatment allegations, involved in 261 court cases, retrieved from the official public judiciary records.	To explore the risk associated with parental mental health problems and types of child maltreatment specifically among parents whose parental rights were terminated.	A cross-sectional design, parents’ mental health problems and the type of child maltreatment they were associated with was selected from the court cases.

Secondly a thematic analysis (Braun and Clarke 2006) was used to reveal similarities and differences in the articles included, concerning sample and phenomenon of interest. The thematic analysis revealed that the articles included different age groups and phenomena of interest. The findings will be presented according to the themes from the thematic analysis under the headings Children and youths, Adults, Forced and arranged marriage, Professionals’ identification of risk of violence, Prevention, and Knowledge gaps. The articles apply different designations to the target group such as intellectual disabilities, intellectual developmental disabilities, learning disability/ties, cognitive disabilities/difficulties/impairment. In Table 1 and in the results, we use the same concepts as the study we refer to.

## Results

### Children and youths

#### Children with and without intellectual disabilities, population studies

Based on a nationally representative sample of sexually abused children in the United States, Helton et al. (2018) found that children with learning difficulties were twice as likely to be sexually abused than children without. Girls with learning difficulties were more than twice as likely to be victims as boys and were also more at risk of experiencing penetrative sexual abuse. The abuse of boys with learning difficulties was to a greater extent non-penetrative, which was similar to abuse of boys without learning difficulties. MacLean et al. (2017) found, in a population-based record-linkage study of all children born in Western Australia between 1990 and 2010, that both suspected and confirmed abuse were reported to a significantly higher degree among children with disabilities than among those without. The risk of maltreatment differed depending on the type of impairment, and children with intellectual disabilities had the highest risk of both suspected and confirmed maltreatment.

#### Children and families

Paquette et al. (2018) reported that children with intellectual disabilities are at higher risk of family violence and of being abused by a parent than other children. There is a higher risk of abuse among children who, in addition to their intellectual disabilities, also have physical disabilities or have self-destructive behaviors. There was a risk of neglect if at least one caregiver had intellectual disabilities. However, parental intellectual disabilities do not predict abuse. Abuse in these families is merely more often detected than in other families due, for example, to overrepresentation in social services. Rather, the socio-demographic characteristics of the family and available social support are associated with the quality of parenting and thus with risks of abuse and violence (Paquette et al. 2018).

This finding is in accordance with those of McConnell et al. (2011) who reviewed over 11,000 investigations carried out because of suspected parental abuse of children and found that 10% of the children had parents with cognitive impairment. Neglect was the most common form of abuse. Parents who were perceived as uncooperative by the professionals were more often reported as neglecting their children than parents who followed directives from the professionals. The researchers discussed whether the lack of cooperation reflects an abusive parenthood or indicates that the parents need support in their parenthood.

Difficulties among parents may also be connected to mental ill health. Vered (2021) examined the relationship between parental mental ill health and the risk that parents abused their children, in a group of parents who had been convicted of child abuse. Children with intellectual disabilities were at higher risk of being abused and neglected and parental mental ill health was a risk factor for abuse. However, there were variations in risk levels between different conditions and gender. A greater risk of child abuse was found if mothers had a mental illness or personality disorder, and children with intellectual disabilities were specifically at risk of child abuse. A mental health diagnosis in the father did however not predict child abuse. This may be a result of mothers more often being the primary caregiver.

Meer and Combrinck (2017) interviewed professionals who described complex and contradictory family relationships, depending on different socio-economic conditions and the social context of the family. Families were described as a place of protection and support for family members with intellectual disabilities. Simultaneously, families could constitute a risk for family members with intellectual disabilities. Their well-being was not always prioritized, and they could be neglected, isolated, and victims of physical and sexual violence. Furthermore, these families could lack support structures, as they were excluded from relatives and networks, due to prejudice associated with intellectual disabilities.

#### Children and youths’ perceptions of risks

A study by Robinson and Graham (2021) included children and youths with intellectual disabilities, and investigated their understanding of risk and safety, and what facilitates and hinders safety in their everyday life. Key themes were; the needs to understand the meaning of safety, how safety was enhanced through familiar contexts with familiar people, having strategies to minimize, handle and avoid risk, opportunities to practice these, having opportunities to learn about safety, and being assured that one is known and valued. Although risk strategies were helpful, they could also result in the participants trying to make themselves invisible, which, according to the researchers, could make them more vulnerable to violence. It was also found that it could be difficult for participants to be heard when they informed adults about their fears, and a limited number of adults listened to them when they felt threatened or frightened.

#### Youths

The studies concerning the risk of violence and abuse among youths (Almack et al. 2009; Carrellas et al. 2021) investigated (1) parents’ perceptions about transition into adulthood, (2) youths in social care system, and (3) risks of being involved in transactional sex.

Parents of children with intellectual disabilities in transition into adulthood perceive accidents, and sexual, emotional, physical, or financial abuse as considerable risks (Almack et al. 2009). Parents struggle with being perceived as overprotective, being unable to “let go”, difficulties to trust that others act in the children’s best interest, and with supporting their children to develop their independence. The parents sensed that their children’s dependence, and their limited opportunity to develop the ability to anticipate and understand the consequences of decisions and behaviors, were considerable risks. There were experiences of insecurity and a sense that societal preventive work against abuse is lacking. This contributes to parents’ perception that they are alone in taking responsibility for their children.

Carrellas et al. (2021) conducted a study concerning youths who transitioned from child welfare to living on their own, which can be particularly risky if there is no support system. The results revealed that 40% of the young participants who had been in child welfare had intellectual disabilities. A higher intellectual ability score was associated with lower risk of engaging in paid sexual activity. The researchers highlight the need for early identification of intellectual disabilities among children in welfare, provision of social support and services, and building community connections during transition to prevent sexual victimization.

### Adults

Studies including adults investigated risks in their everyday life (Prendergast et al. 2015), domestic violence (Fanslow et al. 2021; Lin et al. 2010), exposure to sexual violence and violent crimes (Ledingham et al. 2022; Nixon et al. 2017), the perception of abuse and abusers among persons in the target group (Northway et al. 2013a; 2103b) and ignorance of risks (Kahonde and Johns 2022).

#### Adults and sexual abuse

Adults with intellectual disabilities are more at risk of being exposed to violence and sexual crimes, compared to those without (Majeed-Ariss et al. 2020; Nixon et al. 2017). Women with any type of disabilities reported experiencing sexual violence in their lifetime to a significantly higher degree than women without disabilities (Ledingham et al. 2022). Moreover, women with cognitive or multiple disabilities were significantly more likely to experience either physical or nonphysical force during their first intercourse than women without disabilities.

Nortway et al. (2013a) found that adults with intellectual disabilities need support to disclose abuse and also need ongoing care to address psychological issues. It is further reported that the target group may have difficulties handling and avoiding situations, which exposed them to risks (Kahonde and Johns 2022). For example, it was difficult for them to define what it means to know and trust a person. Some related that they could accompany a person they had just met but would say no to sex. However, they had limited knowledge of what inappropriate touching could be, that it is forbidden for adults to have sex with children, and that perpetrators can be known to victims. Most of the participants could not identify the risks of social media and could not explain the words “rape” or “sexual assault”.

#### Adults and domestic violence

The risk of violence depends on several factors, such as the number of contacts that persons with intellectual disabilities have. The narrative case study of Prendergast et al. (2015) shows that everyday life includes interaction with caregivers, professionals, and relatives which provides opportunities to receive support, but also a risk of violence and abuse.

Domestic violence toward persons with all sorts of disabilities in Taiwan became more frequently reported during 2006-2009 (Lin et al. 2010). The reported prevalence was especially increasing for those with intellectual disabilities, chronic psychosis, and speech disabilities. It should be noted that the differences between groups are small. Nevertheless, the findings point to the need to pay attention to domestic violence towards these vulnerable citizens, prevent it, and provide support. This is in accordance with the study by Fanslow et al. (2021), which found that persons with various forms of disabilities reported higher levels of intimate partner violence than persons without disabilities. More precisely, women with disabilities reported a higher degree of sexual violence in intimate relationships than men, but men with intellectual disabilities were more likely to report physical violence in close relationships than women with intellectual disabilities.

### Forced and arranged marriage

Studies into forced and arranged marriage (Clawson and Fyson 2017; Clawson et al. 2020; McCarthy et al. 2021) have included adults as well as children and youth. Persons with learning disabilities are five times more likely to be forced into marriage than persons without, and males and females are equally vulnerable to forced marriage (Clawson et al. 2020). These studies were conducted in the UK. It should be noted that marriage that includes one party that is unable to give meaningful consent, is considered forced under UK legislation. Consequently, the term forced does not require concrete force or coercion.

The age category in which forced marriage was most frequent was 21–30 years and the second largest age category was 11–20 years. Persons with learning disabilities who were forced into marriage were often under the age of 18 (Clawson and Fyson 2017) The most common reason for forced and arranged marriage was that parents wanted to ensure that someone provided support for the child (Clawson and Fyson 2017; McCarthy et al. 2021). Finding a partner who did not have a learning disability was central for parents, as they were afraid of suffering a double need for care if their child married a person with support needs. It was found that parents were aware that forced marriage may involve abuse. Cultural and religious beliefs and traditions alongside a perception that the child needs to be cared for within the family, and that marriage is a form of normalization, also came forth.

### Professionals’ identification of risk of violence

Nyberg et al. (2021) interviewed professionals who either worked with children with disabilities, or with children exposed to violence. The aim of the study was to identify and validate signs of abuse of children with disabilities. Their participants were asked to rank signs of violence and abuse. The ranking differed between those who worked with children with disabilities and those who worked with children exposed to violence. These results suggest a need to increase the understanding of signs of violence among professionals who work with the target group. Kildahl et al. (2020) reported a case study in which a team of professionals with extensive knowledge and experience of working with persons with intellectual disabilities failed to detect sexual abuse in a young man with intellectual disabilities. They did not ask him about abuse, used no assessment instruments or routines to identify exposure to violence. Retrospectively, they understood that he tried to tell them about the abuse, for example through asking questions about sexuality and leaving pictures with suicidal and sexual content so that the staff could find them. He did not want to return to the place which later turned out to be the place of abuse, and he showed signs of depression. The results show the need for knowledge about abusee among professionals working with the target group, and that symptoms of abuse should be routinely assessed. Moreover, the target group may need referral for special assessment to identify whether they have experienced abuse, so that protection and support can be provided.

### Prevention

#### Prevention – professionals

MacLean et al. (2017) found a need for awareness and competence among professionals regarding the risks of abuse of children with intellectual disabilities. Moreover, parental support is important, as being a parent to a child with disabilities can be stressful and complex. Professionals who do not work directly with the target group, but occasionally meet them, need training to identify the target group so that they can support them in reporting violence, finding treatment, and navigating the legal system. Raising awareness among professionals and parents is important for prevention. Moreover, Robinson and Graham (2021) emphasize that an important part of prevention and protection against abuse is to make persons with intellectual disabilities visible, not only to professionals, but to the entire society.

#### Prevention – the target group

To develop successful preventive measures for children with intellectual disabilities, language and communication difficulties must be acknowledged, which general preventive programs seldom do (Helton et al. 2018). Children with intellectual disabilities need to work with concrete situations, learn one step at a time, repeat what they have learned, and practice what they have learned in real situations. Furthermore, extra time and adaptations such as using pictures and videos ensure that the children will be able to identify risk situations and what strategies they should use to avoid the situation. The same principles could be used when designing programs to prevent violence exposure for youths and adults. Fanslow et al. (2021) reports on a lack of preventive methods and emphasizes the need to design preventive methods for persons with intellectual disabilities.

Nixon et al. (2017) point to the need for risk assessment and the need to consider both individual factors, so that individuals in the target group can be supported to develop their communication skills, and organizational factors, such as assistance in accessing legal support as well as available treatment interventions. Kahonde and Johns (2022) report that persons with intellectual disabilities must receive continuous information about risks of sexual violence, and they suggest that persons with intellectual disabilities should name at least two persons they trust who they can contact if they need help. These findings are also supported by a study by Northway et al (2013b) in which persons with intellectual disabilities stated they need more knowledge and more awareness of personal safety issues and more safety training. This includes having personal alarms, locking the door to the home, telling others where you are and not to be out during the night. The participants in this study also revealed the importance that those who had experiences of some sort of abuse should tell others that they trust to receive support. Other sources of support the participants mentioned were for example “self-help groups, the Internet, therapeutic sessions such as music, and organizations… “ (239).

Programs for preventive measures must take a comprehensive approach and include education about healthy and unhealthy relationships, sexual abuse, and sexuality. Education must use correct terminology, so that the target group can express themselves and be understood when they talk about sexual abuse. Stevens (2012) describes the importance of raising public awareness, changing attitudes about females among boys and men, and developing protective factors at the individual level. For women with intellectual disabilities, sexual health education is proposed to distinguish safe and legal sexual behaviors from abusive and criminal sexual behaviors. Sexual education that promotes sexuality as a part of life, and insight into how sexual situations are negotiated, can be learned alongside the right to say no or yes, and the use of contraceptives.

De Mello et al. (2021) report on experiences of conducting “Dialogic Feminist Gathering (DFG)”, a training intervention for youth with intellectual disabilities aimed at preventing them from being socialized into gender-based violence. The results revealed that the dialog between teachers and students with intellectual disabilities provided possibilities to share experiences of violence and that DFG may protect adolescent girls with intellectual disabilities from relationships that involve sexualized violence. Hughes et al. (2020) have developed “Safety Class”, a program to prevent exposure to violence among adults with intellectual disability. It is an interactive, structured group program conducted over eight sessions focusing on improving participants’ ability to acknowledge safety, build relational skills, and promote safety behaviors with the aim of preventing or reducing violence. The study shows that adults with intellectual disabilities successfully acquired knowledge about safety and became better equipped to recognize unacceptable actions. When persons with intellectual disabilities can identify when they are exposed to abuse, they will more likely report abuse and receive support, thereby reducing the risks.

### Knowledge gaps

Knowledge concerning the risk of family violence toward children with intellectual disabilities seems to be relatively well covered in previous research. Fewer studies have explored the risks in the wider environment: daycare centers, schools, and violence during leisure time. Furthermore, studies focusing on the risk of violence among youths seem to be few, which is unexpected as youth studies in general focus on risks of violence. Other knowledge gaps include the risk of violence between youths with intellectual disabilities. Northway et al. (2013a) but also how to strengthen the awareness among adults with intellectual disabilities of safety issues reported by Northway et al (2013b). Majeed-Ariss et al. (2020) observed a need for research into the relationships between mental health and exposure to abuse. Accordingly, connections between abuse and mental ill-health should be further investigated. Further studies also should include children and youths’ understanding of risk and safety, and what facilitates and hinders safety in their everyday life (Robinson and Graham 2021). No studies concerned risk situations for elderly persons with intellectual disabilities. This group needs to be focused on in future studies. Relatively few studies have the target group as respondents, which means that there is a lack of knowledge based on their perspectives.

The reviewed studies on domestic and intimate partner violence mostly focused on females, and Carrellas et al. (2021) point out that the risk of sexual violence against boys and young men with intellectual disabilities must be investigated. Further knowledge gaps are strategies to handle risks and identify risk factors and situations among children, youth, and adults. There are also knowledge gaps concerning risk assessment and effective interventions to prevent abuse, including to prevent repeated exposure to violence.

The present study also found knowledge gaps concerning ways to identify (i) the target and (ii) violence against them. Moreover, the relative absence of research on preventive measures, and how they can be developed, is a major knowledge gap (Robinson and Graham 2021). Research in this area requires a design that enables follow-up and evaluation and should be accompanied by commitments at the societal level. Kahonde and Johns (2022) and Northway et al (2013b) also state that research must develop preventive measures to protect the target group from violence. An example of attempts to protect the target group from violence is the prevention program “Safety Class”, which seeks to prevent exposure to violence among adults (Hughes et al. 2020). However, a longitudinal randomized controlled study is needed to investigate the effectiveness of the program. In sum, further research is needed to develop appropriate, evidence-based interventions to prevent violence.

## Discussion

The results from this review are based on broad search terms to cover variations of violence, abuse, and risk among all different ages of persons with intellectual disabilities. Violence against persons with intellectual disabilities is known to be a problem worldwide, and there is extensive research that confirms the continuous vulnerability and their risk of exposure to violence and abuse. Few studies have however discussed violence and abuse against youths and elderly with intellectual disabilities. Research about risks for youths (Almack et al. 2009; Carrellas et al. 2021) is less common than research about children and adults. Since the present review included individuals throughout the life course, it adds knowledge about the need for further studies that aim at examining youths and elderly.

Some studies in the present review (McConnell et al. 2011; Paquette et al. 2018; Vered 2021) focused on parents as persecutors and discussed whether parents with intellectual disabilities or mental health issues are at risk of abusing their children. This might not indicate that these parents are more violent than other parents, but rather that they more often are in contact with professionals than other parents. Thereby, child abuse, mostly neglect, is identified to a greater extent among these parents, which is explained by them not receiving information about childcare (Lima et al. 2022). The studies of parental abuse can be related to the life course perspective, and to the family environment as a risk (Meer and Combrinck 2017), revealing the complex and contradictory relationships in families with members who have intellectual disabilities. It should be noted that strained living conditions and poverty may generate violence among parents. Moreover, prejudice about disabilities made relatives withdraw support and even exclude families with members who are intellectually disabled (Meer and Combrinck 2017).

The risk of violence is present in different settings and is difficult for persons with intellectual disabilities to identify, not least because they may lack knowledge about what constitutes a risk. Therefore, the target group needs more information, training, and education (Kahonde and Johns 2022; Northway et al. 2013b; Robinson and Graham 2021). However, education and training should be adapted to the needs of persons with intellectual disabilities and must be age appropriate and evaluated. Furthermore, risks of violence cannot be identified and prevented if professionals do not have sufficient knowledge of violence and about persons with intellectual disabilities (Kildahl et al 2020; Nyberg et al. 2021).

Taken together, the review clarifies that knowledge about violence and intellectual disabilities covers two different professional and organizational fields. Hickson et al. (2013) identified knowledge gaps concerning collaboration and knowledge exchange between professionals specializing in the field of intellectual disabilities and those who meet people exposed to violence. There is a lack of cooperation between professionals in the field of disabilities and professionals in other fields (Didi et al. 2016). This lack might imply that knowledge is not transferred between professional and organizational fields, which means that knowledge about the target group does not always reach professionals who meet them less often. This could concern professionals within legal systems, health care, and those who work in units providing support to victims of violence and abuse. If these professionals cannot identify the target group, appropriate assessment cannot be made and the risks of being exposed to repeated violence and abuse cannot be addressed.

### Limitations of the study

This literature review's aim was to capture a broad life course perspective, considering that the target group's definition differs in the including studies. Instead of limiting the search terms, for the sample only using the search term intellectual disability, we decided to use many different search terms to cover as many studies as possible to be able to answer the research questions. However, this also means the sample in this literature review is heterogeneous rather than homogeneous, which may be seen as a limitation. We also decided to use broad search terms concerning the phenomena of interest, meaning we did not specify different variations of violence. This means that we might have failed to find studies including specific forms of violence.

### Implications for practice

The findings stress the need for collaboration among practitioners working in services for persons with intellectual disabilities, in health care, social service units and legal systems who encounter victims of violence, as well as in crime prevention. Professionals should provide and share knowledge among each other to secure support for the target group. The knowledge gaps point to a need for professionals to use interventions designed for or adapted to meet the target group's needs. The effectiveness of these interventions for identifying both violence (Kildahl et al. 2020) and the target group (Fisher et al. 2012) should be investigated. The research reveals a need to educate the target group about risk and violence, but also to educate professionals about communication with and exposure to violence in the target group (Kahonde and Johns 2022). The need to be able to identify the target group is of particular importance because if the target group cannot be identified, they receive neither support when they have been exposed to violence, nor preventive measures.
